# Spectrochemical differentiation in gestational diabetes mellitus based on attenuated total reflection Fourier-transform infrared (ATR-FTIR) spectroscopy and multivariate analysis

**DOI:** 10.1038/s41598-020-75539-y

**Published:** 2020-11-06

**Authors:** Emanuelly Bernardes-Oliveira, Daniel Lucas Dantas de Freitas, Camilo de Lelis Medeiros de Morais, Maria da Conceição de Mesquita Cornetta, Juliana Dantas de Araújo Santos Camargo, Kassio Michell Gomes de Lima, Janaina Cristiana de Oliveira Crispim

**Affiliations:** 1 Post-Graduate Program in Technological Development and Innovation in Medicines, Federal University of Rio Grande do Norte, Natal, RN 59072-970 Brazil; 2 Biological Chemistry and Chemometrics, Institute of Chemistry, Federal University of Rio Grande do Norte, Natal, RN 59072-970 Brazil; 3 Lancashire Teaching Hospitals NHS Trust, Royal Preston Hospital, Fulwood, Preston, PR2 9HT UK; 4 School of Pharmacy and Biomedical Sciences, University of Central Lancashire, Preston, PR1 2HE UK; 5 Januario Cicco Maternity School, Federal University of Rio Grande do Norte, Natal, RN 59072-970 Brazil

**Keywords:** Biochemistry, Biotechnology, Biomarkers, Endocrinology, Chemistry

## Abstract

Gestational diabetes mellitus (GDM) is a hyperglycaemic imbalance first recognized during pregnancy, and affects up to 22% of pregnancies worldwide, bringing negative maternal–fetal consequences in the short- and long-term. In order to better characterize GDM in pregnant women, 100 blood plasma samples (50 GDM and 50 healthy pregnant control group) were submitted Attenuated Total Reflection Fourier-transform infrared (ATR-FTIR) spectroscopy, using chemometric approaches, including feature selection algorithms associated with discriminant analysis, such as Linear Discriminant Analysis (LDA), Quadratic Discriminant Analysis (QDA) and Support Vector Machines (SVM), analyzed in the biofingerprint region between 1800 and 900 cm^−1^ followed by Savitzky–Golay smoothing, baseline correction and normalization to Amide-I band (~ 1650 cm^−1^). An initial exploratory analysis of the data by Principal Component Analysis (PCA) showed a separation tendency between the two groups, which were then classified by supervised algorithms. Overall, the results obtained by Genetic Algorithm Linear Discriminant Analysis (GA-LDA) were the most satisfactory, with an accuracy, sensitivity and specificity of 100%. The spectral features responsible for group differentiation were attributed mainly to the lipid/protein regions (1462–1747 cm^−1^). These findings demonstrate, for the first time, the potential of ATR-FTIR spectroscopy combined with multivariate analysis as a screening tool for fast and low-cost GDM detection.

## Introduction

Gestational diabetes mellitus (GDM) is a hyperglycaemic metabolic disorder that first appears during pregnancy and does not meet the criteria for manifest diabetes^1^, it is characterized by glucose intolerance or beta cell dysfunction and insulin resistance, and affects up to 22% of all pregnancies worldwide^2^.

One of the protocols that is most used in the diagnosis of GDM follows the recommendations of the American Diabetes Association (ADA)^3^. In addition to hyperglycemia, other glycemic markers have been used for the diagnosis of diabetes mellitus (DM), including fructosamine, glycated albumin, hemoglobin A1c (HbA1c), and 1,5-anhydroglucite, each with its own limitation, if we consider cost for countries in development^4^. Despite this approach, several researchers are looking for new possibilities to identify women at risk for GDM, particularly in the first trimester.

It is known that GDM is considered a risk factor associated with many perinatal morbidities that affect maternal and foetal/neonatal health^1^. GDM promotes increased weight and triglyceride levels, changes in blood pressure, heart problems, induction of caesarean section, and type II diabetes after childbirth in women. For new-borns, the most common risks are weight gain (macrosomia), shoulder dystocia at birth, congenital heart defects, hyperbilirubinemia, polycythemia, respiratory distress and stillbirth, in addition to the risk of developing metabolic syndrome^5,6^.

Individuals with GDM during pregnancy are known to suffer physiological changes, with the appearance of diabetogenic placental hormones (oestrogen and progesterone), placental factors (human placental lactogen), and increased lipids and adipokines including leptin, resistin and visfatin from the first trimester. These contribute to the predisposition of metabolic diseases and insulin resistance, obesity and chronic inflammation capable of releasing different pro-inflammatory cytokines and C-reactive proteins (CRP), especially when these women are obese^7^.

In regard to the contribution of biomolecules in the pathophysiology of GDM, this is not yet well known, however, recent studies have shown that the levels of Growth differentiation factor 15 (GDF15), also known as macrophage inhibitory cytokine-1 (MIC-1), are highly expressed the placenta, and this is identified as a pleiotropic protein that plays key roles in prenatal development, induced by both acute and chronic inflammatory states, acting directly on metabolism of carbohydrates and lipids of GDM women^8,9^. Due to the metabolic impact of GDM during pregnancy, screening and appropriate management of GDM is essential, especially in the first weeks of pregnancy, aiming at improving the quality of prenatal care of these women. The diagnosis of GDM and early intervention is of great significance for reducing short- and long-term consequences for the mothers and new-borns^10^. This is critical in less developed countries, where most pregnant women do not have the opportunity to perform early GDM diagnosis.

Therefore, there is a need for accurate and low-cost techniques for GDM detection. Attenuated total reflection Fourier-transform infrared (ATR-FTIR) spectroscopy can be used to extract spectrochemical information of biological samples, where signals of vibrational motions existing in the chemical bonds of these biomolecules can be captured, hence, generating an important biofingerprint spectrum in the region between 1800 and 900 cm^−1^ where many important biomolecules (DNA/RNA, lipids, proteins and carbohydrates) have contributing metabolic features relating to disease appearance^11^.

Chemometric methods are often employed to analyse complex spectral data acquired with ATR-FTIR spectroscopy. Feature extraction and selection methods, such as principal component analysis (PCA), successive projections algorithm (SPA) and genetic algorithm (GA) can be employed to reduce data complexity and redundant information^12^. PCA is an exploratory analysis algorithm capable of reducing the original data into a low number of principal components (PCs), where each PC represents a piece of the original data variance^11^, while SPA and GA are able to select the most significant wavenumbers from the spectral dataset responsible for class differentiation^13^. These algorithms are commonly associated with linear discriminant analysis (LDA), quadratic discriminant analysis (QDA) and support vector machines (SVM). These classification algorithms are used to build supervised training models which allow us to predict unknown samples based on their spectral response^12^.

ATR-FTIR together with chemometric methods has played an increasingly important role in the field of medical and biological analysis, through quickly detecting pathological conditions, even at very early stages.

Previous studies have demonstrated the importance of using infrared spectroscopy in samples of biological diabetics when analyzing glycation in nail clippings. These studies have shown that ATR-FTIR is sensitive enough to analyze the presence of glucose when compared to the reference population^14^. ATR-FTIR also demonstrated its use in the diagnosis of diseases such as cancer^15^, neurodegenerative diseases^16^, zika and chikungunya^17^ and chronic diseases^18^, as well as in analyzing blood plasma, and managing to separate the disease group from the healthy group, via biomolecules.

## Material and methods

### Study design and population

We performed a case–control study, conducted in a Reference Obstetrics and Gynecology Hospital between January and October 2018. A total of 50 GDM women were recruited, all with single pregnancy at a gestational age of between 12 and 38 weeks. Only participants with complete clinical information were included in the analysis. Subjects were excluded if they had had chronic medical conditions, including hypertension, were declared diabetic (blood glucose ≥ 126 mg/dL), had type 2 diabetes mellitus, and heart or kidney diseases. The study was approved by the Ethics Committee of Federal University of Rio Grande do Norte. Written informed consent was obtained from every participant. All procedures were performed in compliance with the Declaration of Helsinki.

### Clinical measurements

Baseline anthropometric measurements were completed at recruitment using a standardized protocol for BMI classification by week of gestation, the classifications were: underweight, adequate weight, overweight and obesity. Clinical data were collected from medical record reviews. Pregnant women in the GDM group were already diagnosed with blood glucose changes between ≥ 92 mg/dL and < 126 mg/dL during prenatal care, while patients with blood glucose ≥ 126 mg/dL were considered to be declared diabetic, according to the guidelines of the American Diabetes Association (ADA)^3^. These women were given medical nutrition therapy and/or insulin treatment during their antenatal follow-up. The anthropometric, socioepidemiological and metabolic characteristics of GDM and glucose samples were summarized in Table 3.

### Healthy pregnant control group

Fifty healthy pregnant women were enrolled who attended a low-risk maternity hospital. The pregnant women were between 19 and 44 years old, and at a gestational age of between 9 and 39 weeks. The healthy pregnant control group had blood glucose < 92 mg/dL and all underwent fasting glucose testing and oral glucose tolerance test (OGTT) screening at 24–28 weeks to discard GDM.

### Sample collection and determination for analysis with ATR-FTIR

Venous blood samples were collected from participants following an overnight fast 8 h. After 4 h the blood samples were centrifugated at 3600 rpm for 7 min to separate erythrocytes from blood plasma. 100 µL aliquots of plasma were transferred to eppendorf tubes and stored at − 80 °C until ATR-FTIR analysis. The blood plasma glucose levels were determined as described in Table 3.

### ATR-FTIR spectroscopy

The blood plasma samples were thawed at room temperature for 30–40 min, [n = 100 samples (GDM group = 50) and (healthy pregnant control group = 50)], where 10 μL aliquots (in triplicates) were used for analysis. The spectral data were acquired using a IRAffinity-1S FTIR spectrophotometer (Shimadzu Corp., Japan) equipped with an ATR.

The instrument was set up to perform a total of 32 scans with 4 cm^−1^ spectral resolution for both background and sample spectra, recorded rapidly at the range between 4000 and 600 cm^−1^, as described by Santos et al. with some modifications^17^.

### Data analysis

The data analysis was performed in MATLAB R2014b environment version 8.4 (MathWorks, Inc., USA). The raw spectral data was loaded and pre-processed by cutting the biofingerprint region between 1800 and 900 cm^−1^, followed by Savitzky–Golay (SG) smoothing (window of 15 points, 2nd order polynomial fitting), automatic weighted least squares (AWLS) baseline correction and normalisation to the Amide I band (1650 cm^−1^). The data were mean-centred before analysis.

Samples were divided into training (70%), validation (15%) and test (15%) sets for all classification models by applying the Kennard–Stone (KS) algorithm^19^ to the pre-processed spectra. The training set was used in the modelling procedure, the validation set for internal model optimisation, and the test set was only used in the final classification evaluation. Initially, the data were analysed by principal component analysis (PCA). Each PC is composed of scores (variance in sample direction) and loadings (variance in wavenumber direction), where the scores are used to assess similarities/dissimilarities between the samples, and the loadings show the weight of each wavenumber towards the scores pattern. The PCA decomposition of a spectral dataset ${\varvec{X}}$ takes the following form:

$$ {\varvec{X}} = {\varvec{TP}}^{T} + E $$

where ${\varvec{T}}$ is the scores matrix; ${\varvec{P}}$ is the loadings matrix; and ${\varvec{E}}$ is the residual matrix. The PCA scores were used for exploratory analysis of the data, and as input data for supervised classification models: linear discriminant analysis (LDA), quadratic discriminant analysis (QDA), and support vector machines (SVM).

In addition to PCA, the spectral dataset were reduced to a few spectral features by feature selection methods: genetic algorithm (GA) and successive projections algorithm (SPA). These were coupled to LDA, QDA and SVM for classification, and their performances were compared with the PCA-based approaches. GA^20^ is a type of variable selection algorithm that performs this task by mimicking the evolution process, thus recombining and promoting mutations in different subsets of variables until a determined fitness criterion is reached. The goal of this algorithm is to reduce the total number of variables without changing the type of variable, as occurs when using data reduction via PCA. In this case, GA was used with 100 generations and 200 chromosomes each, and mutation and crossover probabilities were set to 10% and 60%, respectively. SPA^21^ also works by reducing the pre-processed spectral data to a low number of variables maintaining the original spectral information. It works with an iterative process by projecting the spectral variables and selecting those which minimise the data collinearity. The optimum number of variables for SPA and GA was determined by the minimum cost function **G** calculated for the validation set as follow^10^:

2 $$ G = \frac{1}{{N_{V} }} \mathop \sum \nolimits_{n = 1}^{{N_{V} }} g_{n} $$

where ${{\varvec{N}}}_{{\varvec{V}}}$ is the number of validation samples and ${{\varvec{g}}}_{{\varvec{n}}}$ is defined as:

3 $$ g_{n} = \frac{{r^{2} \left( {x_{n} ,m_{I\left( n \right)} } \right)}}{{min_{I\left( m \right) \ne I\left( n \right)} r^{2} \left( {X_{n} ,m_{I\left( m \right)} } \right)}} $$

where ${{\varvec{r}}}^{2}({{\varvec{x}}}_{{\varvec{n}}},{{\varvec{m}}}_{{\varvec{I}}\left({\varvec{n}}\right)})$ is the squared Mahalanobis distance between the object ${\text{x}}_{\text{n}}$ (of class ${\text{I}}_{\left( {\text{n}} \right)}$) and the centre of its true class (${\text{m}}_{{{\text{I}}\left( {\text{m}} \right)}}$), and ${\text{r}^{2}}\left( {\text{X}_{\text{n}}},{\text{m}}_{{{\text{I}}\left( {\text{m}} \right)}} \right)$ is the squared Mahalanobis distance between the object ${\text{X}}_{\text{n}}$ and the centre of the closest wrong class (${\text{m}}_{{{\text{I}}\left( {\text{m}} \right)}}$).

Like the PCA scores, the selected wavenumbers by GA and SPA were used as input variables for LDA, QDA and SVM. LDA and QDA are discriminant analysis algorithms based on a Mahalanobis distance calculation between the classes, where LDA assumes classes have similar variance structures, thus, using a pooled covariance matrix for distance calculation; while QDA assumes classes have different variance structures, and thus uses the individual variance–covariance matrix for each class in the distance calculation^22^ SVM is a linear classification algorithm that uses a non-linear step called the kernel transformation^23^. The kernel function (in this case, the radial bases function (RBF)) transforms the input spectral data into a feature space that maximises the margin of separation between the classes. Although more powerful than LDA or QDA for classification, SVM is more susceptible to overfitting^24^.

### Model quality evaluation

Model accuracy, sensitivity and specificity were calculated for the test set in order to evaluate the classification performance and validate the models. The accuracy (AC) represents the total number of samples correctly classified; the sensitivity (SENS) and specificity (SPEC) measure the proportion of positives and negatives that are correctly identified, respectively. These metrics are calculated as follows^25^:

4 $$ {\text{AC }}\left( {\text{\% }} \right) = \left( {\frac{{{\text{TP}} + {\text{TN}}}}{{{\text{TP}} + {\text{FP}} + {\text{TN}} + {\text{FN}}}}} \right) \times 100 $$

5 $$ {\text{SENS }}\left( {\text{\% }} \right) = \left( {\frac{{{\text{TP}}}}{{{\text{TP}} + {\text{FN}}}}} \right) \times 100 $$

6 $$ {\text{SPEC }}\left( {\text{\% }} \right) = \left( {\frac{{{\text{TN}}}}{{{\text{TN}} + {\text{FP}}}}} \right) \times 100 $$

where TP stands for true positive; ***TN*** for true negative; ***FP*** for false positive; and ***FN*** for false negative.

## Results

ATR-FTIR is considered a valuable tool capable of analysing different types of diseases by measuring biological-derived samples. Therefore, we used this technique in order to analyse the specificity, sensitivity and accuracy when differentiating the GDM group.

**Figure 1:**
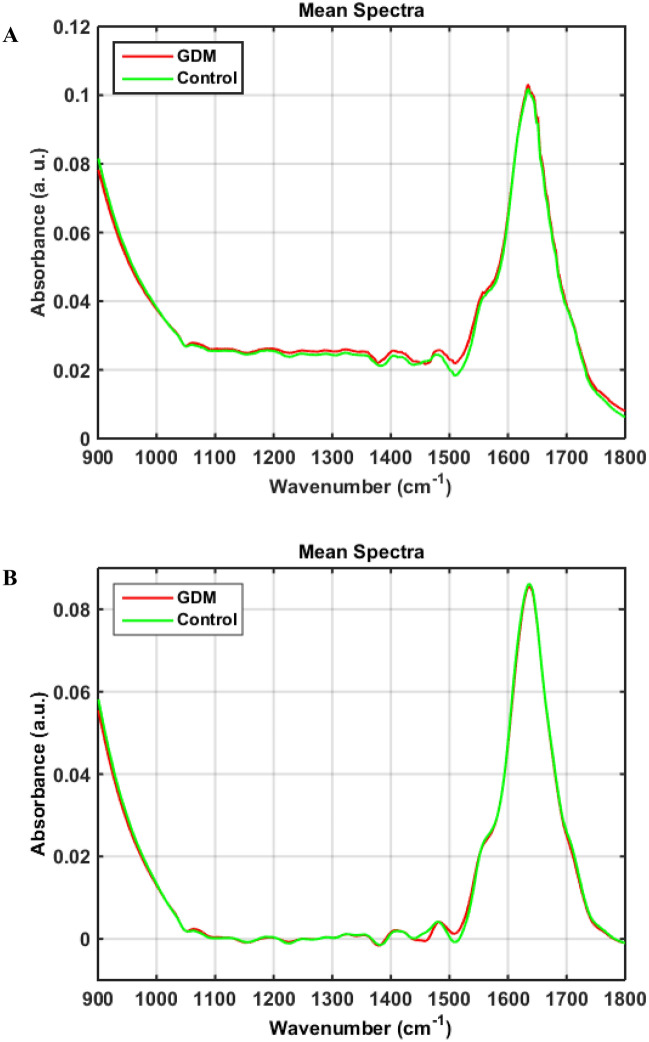
(**A**) Mean raw FTIR spectra for GDM and healthy controls; and (**B**) mean pre-processed spectra (Savitzky–Golay smoothing, baseline correction and normalisation to the Amide I band) for GDM and healthy controls in the biofingerprint region (1800–900 cm^−1^).

The raw ATR-FTIR mean spectra of GDM *vs.* healthy pregnancy control groups are shown in Fig. 1A. The data set consists of 100 samples of blood plasma, 50 samples of GDM group and 50 samples of healthy pregnancy control group. For each sample, the acquisition of 3 spectra was done, giving a total of 300 spectra. In the region of interest between 1800 and 900 cm^−1^, known as the biofingerprint region, some characteristic IR absorption bands can be observed in the spectra, such as the major peaks at ~ 1650 cm^−1^ for Amide I of proteins, as well as methylene groups of lipids at ~ 1750 cm^−1^^26^.

The spectral data were pre-processed by Savitzky–Golay smoothing, baseline correction and normalisation to the Amide I band (~ 1650 cm^−1^) (Fig. 1B). The spectra present strong similarity related to absorption bands, in addition to being highly overlapped, in a way that it becomes difficult to categorise samples only considering the visual spectral information available. In this sense, application of multivariate algorithms is an essential strategy to extract important spectral information, allowing for the discrimination between samples of GDM *vs.* healthy pregnancy control groups based on their pathophysiological condition reflected in the spectral features. Furthermore, variable selection algorithms are powerful tools used to search for biomarkers in blood plasma, allowing less complex models to be obtained.

**Figure 2:**
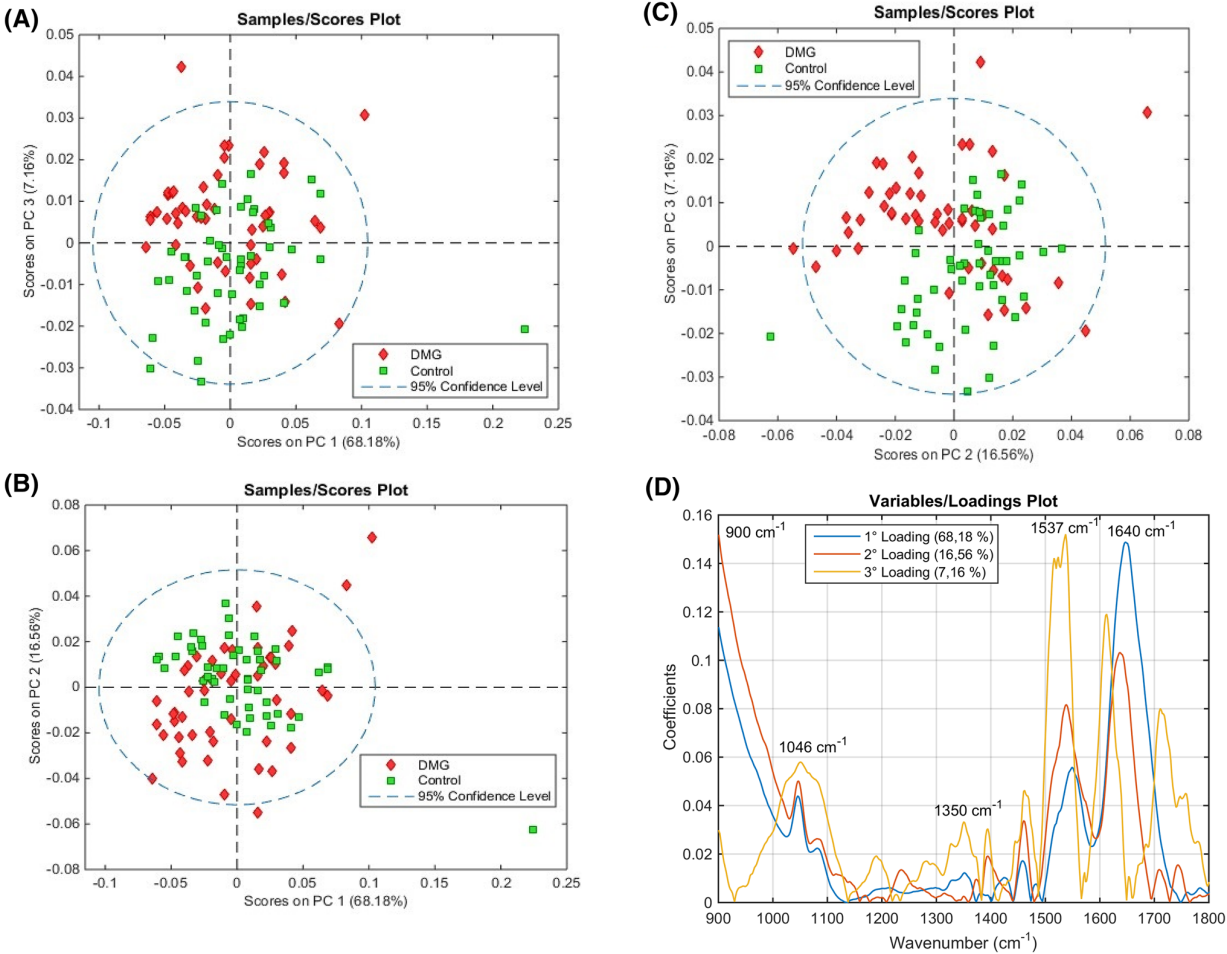
PCA scores plot on (**A**) PC1 *vs.* PC2, (**B**) PC1 *vs.* PC3 and (**C**) PC2 *vs.* PC3. (**D**) PCA loadings on PC1, PC2 and PC3. Percentage inside parenthesis: explained variance.

To predict whether pregnant women are affected by GDM, it is necessary to use chemometric models capable of finding spectral features that differentiate GDM spectra with the healthy pregnancy control group spectra. Initially, a PCA model was performed for exploratory analysis of the data, as shown in Fig. 2. Three principal components (PCs) were used, accounting for > 90% of cumulative explained variance.

The PC1 (68.18% explained variance) *vs.* PC2 (16.56% explained variance) scores plot (Fig. 2A), PC1 (68.18% explained variance) *vs.* PC3 (7.16% explained variance) scores plot (Fig. 2B), and the show some visual distinction between GDM and healthy pregnancy control groups; while the PC2 (16.56% explained variance) *vs.* PC3 (7.16% explained variance) scores plot (Fig. 2C) was much able to efficiently differentiate the sample groups, showing that a low percentage of spectral variance is responsible for class separation.

The PCA loadings are shown in Fig. 2D, where the following spectral features were found to have higher absolute coefficients, thus being responsible for the segregation pattern observed in the PCA scores plot. PC1 and PC2 show very similar loading profiles, with many overlapping bands between 900 to 1500 cm^−1^, and a mirroring profile between 1500 and 1700 cm^−1^; while PC3 shows quite a distinctive loading profile from PC1 and PC2.

**Figure 3:**
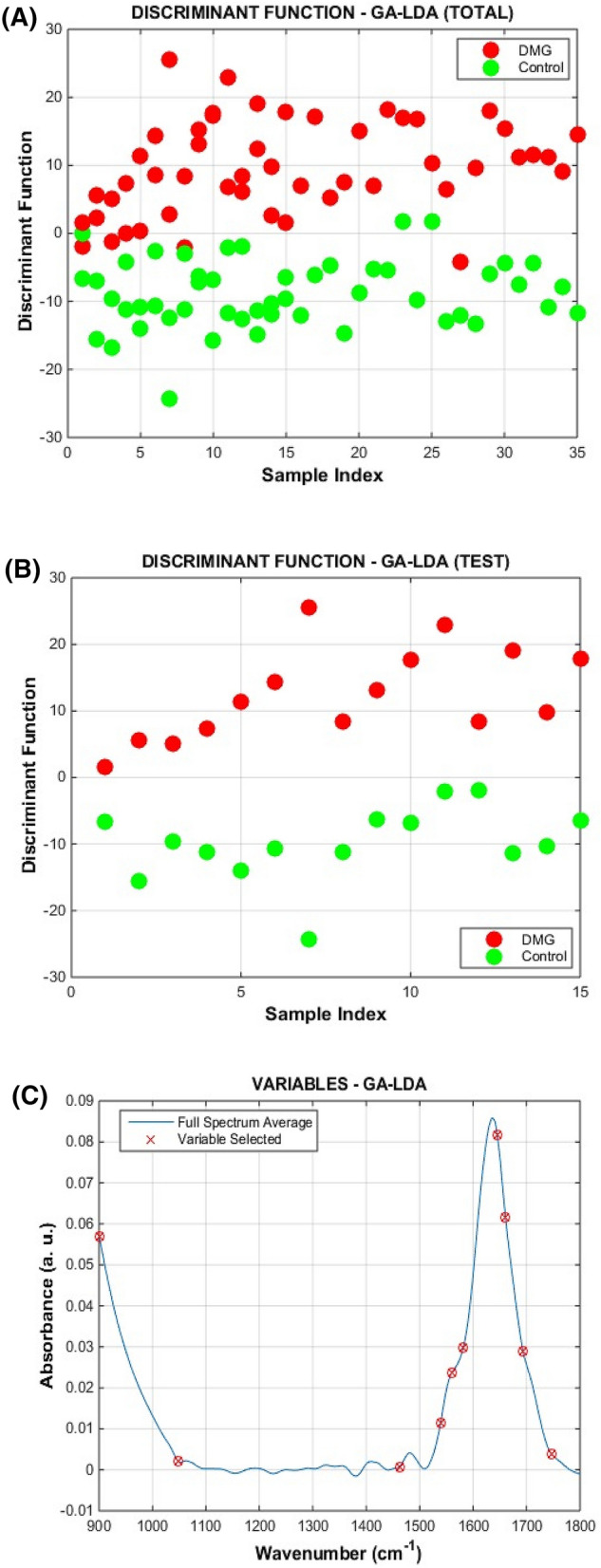
(**A**) GA-LDA discriminant function for all samples (training, validation and test sets); (**B**) GA-LDA discriminant function for the test set only; and (**C**) GA-LDA selected variables.

Supervised classification models were built for systematic discrimination of GDM and healthy pregnancy control groups. For this, the pre-processed spectral data were split into training (70%), validation (15%) and test (15%) sets using the Kennard-Stone (KS) uniform sample selection algorithm. Several classification algorithms were tested (Table 1), where figures of merit were calculated for the test set: accuracy (AC) (percentage of total correct classification), sensitivity (SENS) (percentage of correct classification for the GDM group), and specificity (SPEC) (percentage of correct classification for the healthy pregnancy control group). The genetic algorithm linear discriminant analysis (GA-LDA) model achieved the best classification results, with 100% accuracy, sensitivity and specificity for the test set. GA-LDA Fisher’s discriminant scores (Fig. 3A,B) show an almost complete separation for all samples (training, validation and test sets) (Fig. 3A), and a perfect separation for the test samples (Fig. 3B). Where GA-LDA selected 10 spectral wavenumbers which were responsible for group differentiation, principally associated with the regions for water (901; 1047 cm^−1^) and lipid/protein regions (1462; 1539; 1560; 1582; 1645; 1661; 1693; 1747 cm^−1^) (Fig. 3C). The tentative biochemical assignments of these variables based on Movasaghi et al.^26^ are shown in Table 2.

**Table 1:** Quality parameters for the test set.

Parameter	PCA			SPA			GA		
	LDA	QDA	SVM	LDA	QDA	SVM	LDA	QDA	SVM
AC (%)	83.3	86.7	90.0	90.0	83.3	90.0	100	96.7	86.7
SENS (%)	80.0	100	80.0	93.3	100	80.0	100	100	73.3
SPEC (%)	86.7	73.3	100	86.7	66.7	100	100	93.3	100

*AC* accuracy, *SENS* sensitivity, *SPEC* specificity.

**Table 2:** Selected wavenumbers by the GA-LDA to distinguish GDM and controls samples.

Selected wavenumber (cm^−1^)	Tentative assignment
901	Phosphodiester stretching bands region (for absorbances due to collagen and glycogen)
1047	Glycogen band (due to OH stretching coupled with bending)
1462	CH2 scissoring mode of the acyl chain of lipid
1539	Protein amide II absorption- predominately b-sheet of amide II
1560	Ring base mode
1582	Ring C–C stretch
1645	Amide I
1661	Amide I
1693	High frequency vibration of an antiparallel β-sheet of Amide I
1747	ν(C=O) (polysaccharides, pectin)

While still analyzing the characteristics of both groups, in the present study it was possible to verify some differences in relation to demographic, clinical and obstetric data, as shown in Table 3. Most pregnant women with GDM were older and had previous pregnancies when compared to the healthy pregnancy control group (p < 0.05). When analyzing fasting blood glucose, the GDM group was statistically significant when compared to the healthy pregnancy control group (p < 0.05). The mean BMI of the GDM group was higher (30.78 ± 5.00), compared to healthy pregnancy control group (28.24 ± 4.09), and they presented obesity or were overweight (p < 0.05).

**Table 3:** Demographic factors, clinical and obstetric history of pregnant women with and without diagnosis of GDM.

Variables	Group		p value^a^	Total
	GDM	Control		
N, %	50 (50.0%)	50 (50.0%)		100 (100.0%)
Age, years	32 (28–35)	28 (24–35)	**0.046**	31 (25–35)
Age (≥ 35 anos), n (%)	13 (26.0%)	13 (26.0%)	1.000	26 (26.0%)
Fasting blood glucose, mg/dl	98 (95–107)	79 (73–87)	**p < 0.01**	92 (79–98)
BMI, kg/m	30.78 ± 5.00	28.24 ± 4.09	**0.006**	29.51 ± 4.72
**BMI, n (%)**				
Suitable	8 (16.0%)	25 (50.0%)	**0.002**	33 (33.0%)
Low weight	3 (6.0%)	3 (6.0%)		6 (6.0%)
Overweight	21 (42.0%)	15 (30.0%)		36 (36.0%)
Obesity	18 (36.0%)	7 (14.0%)		25 (25.0%)
Obesity or overweight, n (%)	39 (78.0%)	22 (44.0%)	**p < 0.01**	61 (61.0%)
**Marital status, n (%)**				
Single or divorced	19 (38.0%)	35 (70.0%)	**0.001**	54 (54.0%)
Married or stable union	31 (62.0%)	15 (30.0%)		46 (46.0%)
**Has children, n (%)**				
Yes	40 (80.0%)	29 (58.0%)	**0.017**	69 (69.0%)
No	10 (20.0%)	21 (42.0%)		31 (31.0%)
Number of children	1 (1–2)	1 (0–2)	0.107	1 (0–2)
Had previous pregnancy, n (%)	49 (98.0%)	48 (96.0%)	1.000	97 (97.0%)
Previous pregnancies, qty	3 (2–4)	2 (1–3)	0.110	2 (1–3)
Miscarriage History, n (%)	19 (38.0%)	18 (36.0%)	0.836	37 (37.0%)
**Last delivery type, n (%)**				
Cesarean	12 (24.0%)	14 (28.0%)	0.100	26 (26.0%)
Vaginal	28 (56.0%)	18 (36.0%)		46 (46.0%)
First birth	10 (20.0%)	18 (36.0%)		28 (28.0%)
Own GDM history, n (%)	2 (4.0%)	1 (2.0%)	1.000	3 (3.0%)
Family history of GDM, n (%)	31 (62.0%)	30 (60.0%)	0.838	61 (61.0%)
History of disease in previous pregnancy, n (%)	11 (22.0%)	7 (14.0%)	0.298	18 (18.0%)

Continuous data are expressed as Mean ± Standard deviation/median and 25th and 75th percentiles.

Categorical data are expressed as absolute (n) and relative (%) frequency.

Values in bold indicate significance at p < 0.05.

*GDM* Gestational diabetes mellitus, *qty.* quantity, *BMI* Body Mass Index.

^a^Significance of difference between groups by Student's t-test or Mann–Whitney U test (continuous variables) or Pearson’s chi-square test or Fisher’s test (categorical variables).

## Discussion

The development of a novel tool for the diagnosis of different diseases is extremely important, principally when they affect women during pregnancy, as is the case with GDM which is capable of harming both the mother and the fetus.

ATR-FTIR is considered a powerful tool, as it analyzes different biological structures based on spectral analysis, proving to be of great use to health clinical, promoting future perspectives through technological advances^11^.

In our study, blood plasma from 100 pregnant women (50 GDM and 50 healthy control group) was analyzed by ATR-FTIR spectroscopy, in order to predict GDM group based on their samples’ spectrochemical profile. Our data showed that unsupervised model PCA was able to show a discriminating pattern between the groups, generating better scores between the PCs (PC2 *vs.* PC3). In PC3, the main difference is the amount of protein versus water. The negative loading appears around 1635 cm^−1^ (water band). This appears oppositely correlated with the Amide II indicating a difference in the protein/water ratio between the two groupings. PC2 and PC3 show a great scores difference between the samples groups, indicating their respective loadings on PC1 and PC2 can be used to identify spectral markers associated with class differences. The spectral regions around 1640 cm^−1^, near the water band, showed one of the highest absolute loadings indicating that water is a discriminating feature between the samples. However, Caixeta et al.^27^, when analyzing saliva samples of male wistar rats with DM (treated with insulin), pre-diabetic and healthy, demonstrated the applicability of the ATR-FTIR associated with PCA-LDA, where it was able to generate six PCs, demonstrating the effectiveness of using mathematical algorithms in monitoring DM. Moreover, in a recent study analyzing peripheral blood samples from pre-diabetic patients, a response was found to glucose levels when using ATR-FTIR and PCA combined with eXtreme Gradient Boosting (XGBoost) generating the model SG-PCA-XGBoost, which was able to differentiate from healthy people^18^.

When we used different supervised models, GA-LDA was the best classification model that systematically distinguished GDM samples from controls. GA-LDA is a powerful feature selection algorithm based on iterative combinations inspired by Mendelian genetics, where the fittest variables (wavenumbers) that maximize class separation are selected^13^. It commonly outperforms feature extraction methods such as PCA^28^. However, there are few studies that address the use of the ATR-FTIR tool in diabetes, and fewer with GDM. Until this moment, no study has analyzed blood plasma samples from pregnant women with GDM in GA-LDA models. This demonstrates the innovation of this model in the prediction of GDM, and confirms that GA-LDA is an excellent classification algorithm for samples of blood plasma of pregnant women, playing a fundamental role during prenatal care, assisting in diagnosis and monitoring.

Although many studies on the pathophysiology of GDM have been conducted, the potential of biomarkers in its development remains unclear. In our study it was possible to verify that the selected wavenumbers by GA-LDA were responsible for group separation, according to the biomolecule regions referring to lipid and protein/water ratio. This information combined with the GA-LDA selected wavenumbers at 1046 cm^−1^, 1537 cm^−1^ and 1640 cm^−1^ indicate that some relation between water and protein levels is a discriminant factor between the groups.

However, GDM emerges as a disorder of insulin-dependent, where metabolomic pathways are relevant to lipid and amino acid metabolisms, as well as bile acids and abnormal protein turnover^29^. Promotion of oxidation of protein intensifies during GDM, in which the hyperglycemic state causes protein hydroperoxides, protein carbonyls, C-reactive protein and glycated hemoglobin (HbA1c). In addition to this, it is considered an important mediator of adipocyte disorders, intensifying the inflammatory response and contributing to the complications of diabetes^30^.

To reinforce our data and assessment of the associated factors with GDM, we can observe that there is an increase in BMI, one of the precursors for insulin resistance, since during obesity there is an increase in lipids and there is the release of inflammatory cytokines. In addition, we emphasize that maternal age and obesity are factors that can directly interfere with pregnancy, contributing to the development of GDM.

## Conclusions

According to the results of the present study, blood plasma samples from pregnant women with GDM could rapidly be differentiated from our healthy pregnant control group based on their sample FTIR spectra, where a chemometric model by means of the GA-LDA algorithm, was able to distinguish between GDM and healthy pregnant control group with 100% accuracy, sensitivity and specificity in an external test set.
